# Neurological Aspects of SARS-CoV-2 Infection: Mechanisms and Manifestations

**DOI:** 10.3389/fneur.2020.01039

**Published:** 2020-09-04

**Authors:** Parménides Guadarrama-Ortiz, José Alberto Choreño-Parra, Claudia Marisol Sánchez-Martínez, Francisco Javier Pacheco-Sánchez, Alberto Iván Rodríguez-Nava, Gabriela García-Quintero

**Affiliations:** ^1^Departament of Neurosurgery, Centro Especializado en Neurocirugía y Neurociencias México (CENNM), Mexico City, Mexico; ^2^Escuela Nacional de Ciencias Biológicas, Instituto Politécnico Nacional, Mexico City, Mexico; ^3^Internado Medico de Pregrado, Centro Especializado en Neurocirugía y Neurociencias México (CENNM), Mexico City, Mexico; ^4^Escuela Nacional de Medicina y Homeopatía, Instituto Politécnico Nacional, Mexico City, Mexico

**Keywords:** coronavirus, COVID-19, SARS-CoV-2, severe acute respiratory syndrome, pneumonia

## Abstract

The human infection of the novel severe acute respiratory syndrome coronavirus 2 (SARS-CoV-2) is a public health emergency of international concern that has caused more than 16.8 million new cases and 662,000 deaths as of July 30, 2020. Although coronavirus disease 2019 (COVID-19), which is associated with this virus, mainly affects the lungs, recent evidence from clinical and pathological studies indicates that this pathogen has a broad infective ability to spread to extrapulmonary tissues, causing multiorgan failure in severely ill patients. In this regard, there is increasing preoccupation with the neuroinvasive potential of SARS-CoV-2 due to the observation of neurological manifestations in COVID-19 patients. This concern is also supported by the neurotropism previously documented in other human coronaviruses, including the 2002–2003 SARS-CoV-1 outbreak. Hence, in the current review article, we aimed to summarize the spectrum of neurological findings associated with COVID-19, which include signs of peripheral neuropathy, myopathy, olfactory dysfunction, meningoencephalitis, Guillain-Barré syndrome, and neuropsychiatric disorders. Furthermore, we analyze the mechanisms underlying such neurological sequela and discuss possible therapeutics for patients with neurological findings associated with COVID-19. Finally, we describe the host- and pathogen-specific factors that determine the tissue tropism of SARS-CoV-2 and possible routes employed by the virus to invade the nervous system from a pathophysiological and molecular perspective. In this manner, the current manuscript contributes to increasing the current understanding of the neurological aspects of COVID-19 and the impact of the current pandemic on the neurology field.

## Introduction

The novel severe acute respiratory syndrome coronavirus 2 (SARS-CoV-2) originated in Wuhan, China (1), in December 2019, and it has engulfed the world in an unprecedented global pandemic. The coronavirus disease 2019 (COVID-19) associated with this virus has caused more than 16.8 million new cases and 662,000 deaths as of July 30, 2020 (2), generating a high burden of disease that has exceeded the assistance capacities of several healthcare systems around the world. The clinical characteristics of patients with COVID-19 are similar to those observed during the outbreak of SARS-CoV-1, which emerged in 2002–2003, causing more than 8,000 confirmed cases and ~800 deaths (3). As such, the human infection with SARS-CoV-2 mainly affects the lower respiratory tract, causing mild to moderate respiratory symptoms in about 85% of patients with COVID-19 (4–6), including fever, headache, fatigue, myalgia, dry cough, and diarrhea. Most symptomatic individuals are men in their fifth and sixth decades of life that attend medical centers after an incubation period of about 4 to 5 days (4–7). Another 15% of patients present moderately severe forms of the disease manifested as pneumonia of atypical features in radiological studies of the lung, such as bilateral multi-lobe consolidations and ground-glass opacities (8). Finally, in 5 to 30% of COVID-19 patients, the virus causes severe acute respiratory syndrome (SARS), which is characterized by profound respiratory distress that obligates the establishment of intensive life support interventions, such as intubation and mechanical ventilation (4–6). Initial reports estimated mortality rates of about 2 to 4%. However, new analyses indicate a higher lethality of COVID-19 (9), especially among individuals older than 65 years, and patients with comorbidities (4–6). Unfortunately, the risk of a fatal outcome is disproportionally higher among patients requiring mechanical ventilation, with a mortality rate close to 80% (7).

The rapid transmission of SARS-CoV-2 and the increasing number of positive cases reported around the world have overcome the resources of the healthcare systems in different regions. This has significantly impacted all areas of medicine, especially those directly related to the management of severe respiratory infections, such as pneumology and critical care medicine. Nonetheless, SARS-CoV-2 has the potential to spread to different extrapulmonary tissues, and, in some of the most severe cases, the infection can progress to multiorgan failure (5). Therefore, all healthcare providers from any area of medicine must acquire adequate knowledge of the principal characteristics of COVID-19. Currently, there is increasing preoccupation about the potential capacity of SARS-CoV-2 to invade the central nervous system (CNS) and the peripheral nervous system (PNS). These concerns are based on recent observations in individuals infected with SARS-CoV-2 that present neurological findings (10). A better understanding of the mechanisms underlying the neurologic sequela of patients with COVID-19 is urgent to discover novel targets for therapeutics development. In the current review article, we therefore provide an overview of the spectrum of neurological manifestations of COVID-19. Additionally, we analyze host- and pathogen-specific factors that determine the tissue tropism of SARS-CoV-2 and discuss the possible routes employed by the virus to invade the nervous system. Furthermore, we propose possible therapeutics for the neurologic complications of COVID-19.

## SARS-CoV-2 Biology

### Virology

SARS-CoV-2 is a novel member of the group of human coronaviruses (HCoVs), which is constituted of HCoV-229E, HCoV-NL63, HCoV-HKU1, HCoV-OC43, SARS-CoV, and MERS-CoV (11). These are RNA single-stranded viruses belonging to the Coronaviridae family. Some of these pathogens have caused a variety of respiratory diseases in the past. For instance, SARS-CoV-1 infected more than 8,000 individuals around the world (3). Also, the human coronavirus related to the respiratory syndrome of the Middle East (MERS-CoV), which emerged in Saudi Arabia in 2012 and caused high mortality rates among infected people (12, 13).

Based on sequence comparisons of viral genomes, HCoVs are grouped into four genera: alpha, beta, gamma, and delta coronaviruses. SARS-CoV-2 is a beta coronavirus genetically related to another bat coronavirus named BatCoV RaTG13 as well as SARS-CoV-1 (14, 15). Furthermore, SARS-CoV-2 also shares its genetic identity with coronaviruses isolated from pangolins (16, 17). Hence, it is believed that COVID-19 is a zoonotic disease that originated from bats or pangolins. The genome of SARS-CoV-2 consists of a single RNA strand of 29.903 bp that codifies for the replicase-transcriptase, as well as for the structural proteins spike (S), envelope (E), membrane (M), and nucleocapsid (N) (15).

### Mechanism of Infection and Determinants of SARS-CoV-2 Tropism

The initial step of SARS-CoV-2 infection is the recognition of its receptors on the surface of host cells. This step is mediated by the viral spike (S) protein, which recognizes the human receptor angiotensin I-converting enzyme 2 (ACE2), the same receptor for the S protein of SARS-CoV-1 (18–20). This protein owns two functional domains: the S1 domain contains the receptor-binding domain (RBD), which attaches to ACE2, whereas the S2 domain mediates the fusion of the viral and host cell membranes (20). Therefore, the organ distribution of the ACE2 receptor is a crucial determinant of the virus infectivity and tropism. A second step determinant in the infection process of SARS-CoV-2 is the activation of the S protein. This process is mediated by different host proteases, which execute the cleavage of the molecule at the S1/S2 and S'2 sites. This protein processing allows the complete activity of the S2 domain and the fusion of the viral and cellular membranes. For this purpose, and as in the case of SARS-CoV-1, SARS-CoV-2 uses the transmembrane serine protease 2 (TMPRSS2) (19, 21, 22). Interestingly, the proteases TMPRSS4 and cathepsin L also promote SARS-CoV-2 infection of human small intestinal enterocytes and 293/hACE2 cells (23, 24). Hence, the tissue patterns of expression of TMPRSS2, TMPRSS4, and cathepsin L is another decisive factor that determines the tropism of the virus, and, indeed, some drugs that inhibit the activity of these proteases are now proposed as potential therapeutic agents to prevent and treat COVID-19 (19, 23).

**Figure 1:**
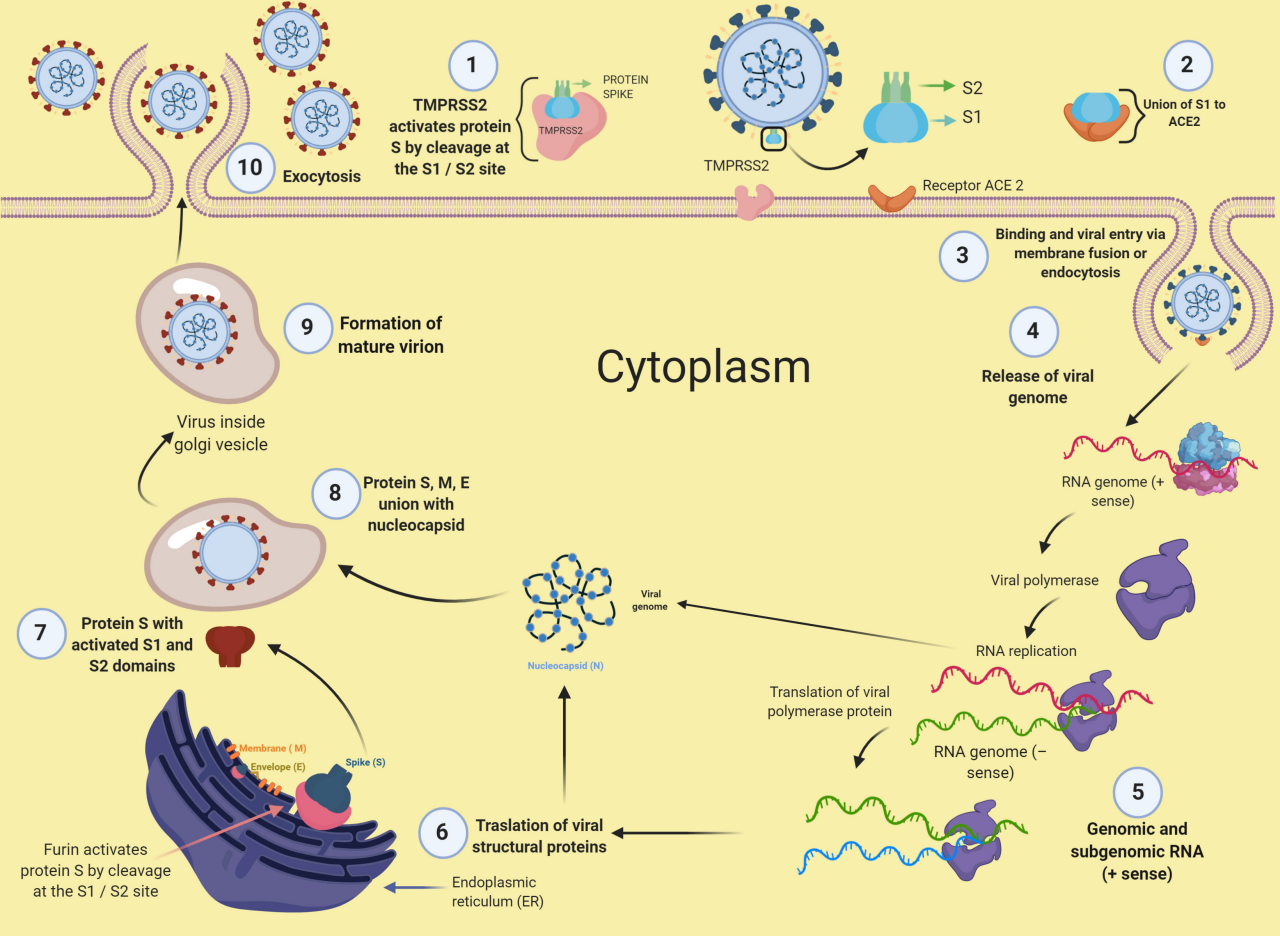
Mechanisms of infection of SARS-CoV-2. This figure was created with *BioRender*.

Other factors implicated in the process of SARS-CoV-2 infection include the phosphatidylinositol 3-phosphate 5-kinase (PIKfyve) (23). This enzyme mediates the production of phosphatidylinositol-3,5-bisphosphate [PI(3,5)P2], a phosphoinositide that participates in the maturation process of endosomes. Treatment with apilimod, a potent inhibitor for PIKfyve, reduced the infectivity of SARS-CoV-2 and could be a novel candidate for therapeutic applications. After the entry into host cells, viral replication begins with the translation of the replicase-polymerase gene and the assembly of the replication-transcription complex. This complex subsequently transcribes the genomic regions that codify for structural proteins. New virions are assembled in the endoplasmic reticulum and Golgi apparatus to finally egress from the cell (11). A particular feature of SARS-CoV-2 is that it possesses a polybasic furin cleavage sequence (PRRA) in the S1/S2 site, which is absent in other close related coronaviruses (14, 20). This inserted furin cleavage sequence is processed at the Golgi apparatus during the biosynthesis of the S protein of novel virions inside the host infected cells (20). The novel virions of SARS-CoV-2 may thus contain an S protein primed and ready to infect any other cells expressing the ACE2 receptor, with no further requirement of TMPRSS2 activity. As virtually all cells express furin under normal conditions, the inserted furin cleavage sequence may expand the transmissibility and tissue tropism of SARS-CoV-2. Figure 1 illustrates the process of SARS-CoV-2 infection.

### The Immune Response Against SARS-CoV-2

SARS-CoV-2 elicits an exuberant immune response characterized by a dysregulated production of soluble immune mediators. This phenomenon has been called a “cytokine storm” and is responsible for mediating tissue damage in patients with COVID-19 that progress to severe illness (25–28). The immune receptors that recognize the viral infection and initiate the immune responses against SARS-CoV-2 are unknown. As this virus is genetically related to SARS-CoV-1, it is presumed that both viruses share mechanisms of infection. In this sense, SARS-CoV-1 is recognized by the toll-like receptors (TLR) TLR3 and TLR4, which induce an immune reaction via MyD88 and TRIF pathways (29, 30). Furthermore, SARS-CoV-1 triggers the production of IL-1β through the activation of the inflammasome (31). In this regard, the activation of the inflammasome is also possible to occur during SARS-CoV-2 infection, as high levels of IL-1β have been observed in COVID-19 patients (32). Other immune mediators that are exaggeratedly produced in response to SARS-CoV-2 include IL2, IL-6, IL7, IL10, G-SCF, CXCL10, MCP-1, MIP-1A, and TNFα (5, 28, 33). From these, IL-1β, IL-6, CXCL10, and TNFα are the cytokines with a higher capacity to generate tissue damage in several organs, including the CNS, due to their pro-inflammatory properties. For instance, IL-1β and IL-6 have been implicated in neurotoxicity associated with chimeric antigen receptor (CAR) T cell therapy in patients with hematological malignancies (34, 35). These cytokines possess detrimental effects on endothelial function at several vascular niches, which may be implicated in the pathophysiology of the neurological complications of COVID-19, as discussed later.

Notably, despite the dysregulated production of immune mediators, an ample range of immune cell subtypes are depleted from the circulation of patients with severe SARS-CoV-2 infection. These cells include monocytes, dendritic cells, CD4+ and CD8+ T cells, and NK cells (36). Furthermore, the few adaptive lymphocytes that remain in the blood express markers of functional exhaustion (37). These data suggest that severe COVID-19 is a state of immunosuppression similar to the known sepsis-induced immunosuppression (38). However, it is also possible that the robust recruitment of functional immune cells to the sites of SARS-CoV-2 infection may explain the leukopenia observed during COVID-19.

## Mechanism of SARS-CoV-2 Entry Into the CNS

### Expression of SARS-CoV-2 Viral Entry Factors in the CNS

Several HCoVs, including SARS-CoV-1, have the potential to infect different human tissues and organs (39, 40). Similarly, SARS-CoV-2 may also spread to several extrapulmonary tissues, including the CNS. Although recent analyses of autopsy specimens from patients with COVID-19 have not explored the presence of this virus in the brain (41), the neurological manifestations observed among individuals with COVID-19 (10) and the isolation of other human coronaviruses from neurological specimens support the notion of a possible neurotropism of SARS-CoV-2 (42–47). The neurotropism of other HCoVs has been extensively revised elsewhere (48).

As mentioned before, the patterns of expression of ACE2, TMPRSS2, TMPRSS4, furin, cathepsin L, and other entry factors used by SARS-CoV-2 for infection determine the tropism of the virus. The ACE2 receptor is highly expressed in cells of the alveolar epithelium (49, 50), which explains the vulnerability of the lungs to infection. The expression of ACE2 has also been found in other tissues, including the oral mucosa, endothelium, heart, kidney, lymphoid organs, testis, gut, and urinary tract (49–52). Nonetheless, distribution of TMPRSS2 has been assessed in a limited variety of human tissues, showing high expression in prostate cells, respiratory epithelial cells, salivary gland, kidney, liver, stomach, small intestine, and colon (53–55). TMPRSS2 is regulated by androgens (53), which may explain the higher susceptibility of men to suffer from severe forms of COVID-19. Few studies have analyzed the expression of TMPRSS4 and cathepsin L in healthy human organs. According to the Human Protein Atlas (https://www.proteinatlas.org/) dataset, TMPRSS4 is present in the cerebral cortex, hippocampus, caudate, thyroid gland, adrenal gland, nasopharynx, bronchi, lung, stomach, duodenum, colon, rectum, gallbladder, pancreas, and genitourinary tract. Meanwhile, cathepsin L shows medium expression in lung and liver, and low expression at bronchi, salivary gland, liver, kidney, pancreas, and genitourinary tract.

The expression in the human body of the known genes mediating the entry of SARS-CoV-2 into human cells coincides with the multiorgan pattern of COVID-19 manifestations. Nonetheless, the presence of such factors is low in the CNS under normal conditions. This may be in part because previous studies have only analyzed the bulk organ gene expression patterns of ACE2 and TMPRSS2. More recently, a comprehensive analysis of several single-cell RNA-seq databases showed that there are dual-positive ACE2+TMPRSS2+ cells in tissues beyond the respiratory system, including oligodendrocytes in the brain, and inhibitory enteric neurons (56). Furthermore, ACE2+CTSL+ cells were enriched in the olfactory epithelium. These data support some of the possible routes of SARS-CoV-2 entry into the CNS discussed below.

### Routes of SARS-CoV-2 Entry Into the CNS

The mechanisms employed by SARS-CoV-2 to infect the nervous system are unknown. Currently, the hypotheses about the routes of viral entry into the CNS rely on previous observations made in experimental studies of SARS-CoV-1 infection. One hypothesis proposes that SARS-CoV-2 invades the brain by breaching the blood-brain barrier (BBB). Evidence in favor of this infection mechanism includes the high expression of the ACE2 receptor in endothelial cells of blood vessels (49, 50). The virus may therefore infect endothelial cells of the brain vasculature in the first term, and it may then spread to the surrounding ACE2+TMPRSS2+ oligodendrocytes and, finally, to the neurons. This would explain why SARS-CoV-1 has been observed inside neurons even when they have a mild expression of ACE2 under normal conditions (39, 40, 57). Despite this, SARS-CoV-1 has not been isolated from or observed inside endothelial cells (58). Conversely, the high concentrations of pro-inflammatory cytokines in the systemic circulation of patients with severe forms of COVID-19 might induce structural and functional alterations of the BBB (5, 28, 59). In this sense, it is well-known that different inflammatory mediators have detrimental effects on BBB integrity, increasing its permeability to neurotoxic molecules and immune cells (60). SARS-CoV-2 could thus gain access to the CNS directly through a paracellular route or within the immune cells, a mechanism that has been called a “trojan horse”(61). It might be feasible for this mechanism to occur during SARS-CoV-2 infection since the previous SARS-CoV-1 has also been observed inside different leukocyte subsets (40).

Secondly, the virus could enter the CNS through the olfactory epithelium, crossing the cribriform plate of the ethmoid bone and reaching the olfactory bulb from which it could spread to different areas of the brain (62). This route was demonstrated in mice intranasally inoculated with SARS-CoV-1, among which a rapid viral spread from the olfactory bulb to the brain stem was observed. This exposure to the virus caused high lethality among infected animals due to the occurrence of neuronal death in the respiratory centers of the brain stem (63). Similar results were observed in another animal model of CNS infection with the HCoV-OC43 coronavirus (64). In humans, some studies demonstrated olfactory neuropathy in patients with SARS-CoV-1 infection (65). Likewise, recent investigations have shown that the olfactory epithelium is enriched with ACE2+CTSL+ cells (56). Moreover, hyposmia and anosmia are frequent manifestations of COVID-19 (10, 66), suggesting that SARS-CoV-2 might also infect the olfactory bulb in COVID-19 patients. In light of these findings, some researchers have proposed that the neuroinvasive potential of SARS-CoV-2 could contribute to the respiratory failure observed in patients with severe COVID-19 (67).

**Figure 2:**
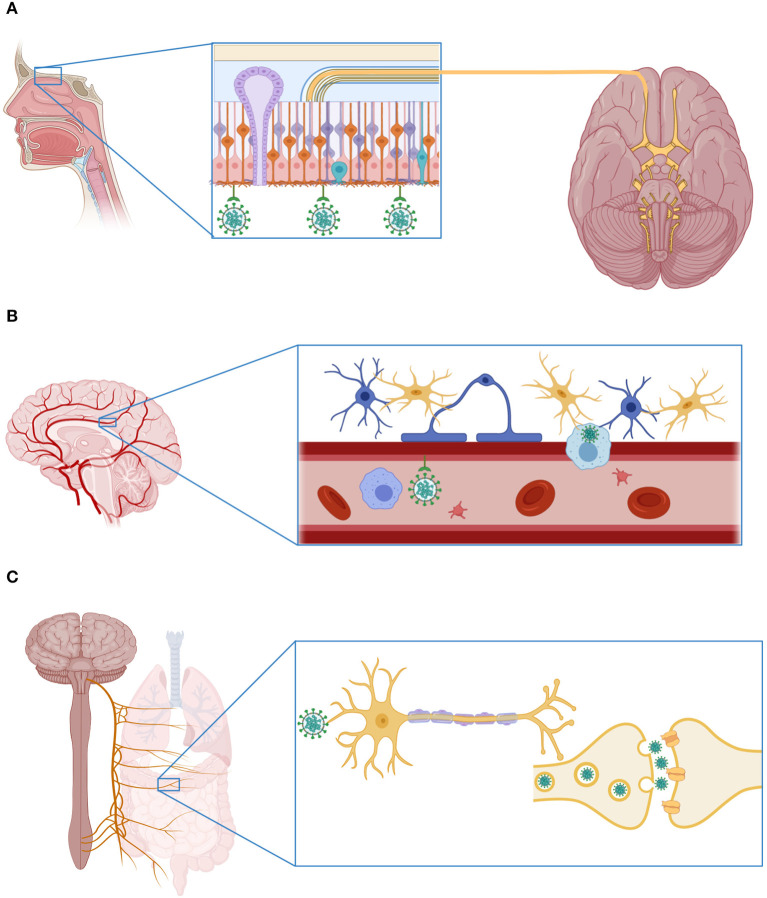
Mechanisms of SARS-CoV-2 invasion to the central nervous system. **(A)** SARS-CoV-2 can enter the CNS through the olfactory bulb. Indeed, recent investigations have shown that the olfactory epithelium is enriched with cells that express the receptor ACE2 and the protease cathepsin L. **(B)** The virus can also infect the CNS via the hematogenous route, attaching to the ACE2 receptor expressed in endothelial cells of the cerebral blood vessels, or inside an immune cell. **(C)** Finally, SARS-CoV-2 can infect the nerve terminals of the vagus nerve located in the respiratory system and the gastrointestinal tract. From these sites, the virus can reach the CNS using the mechanisms of retrograde neuronal protein transport and transsynaptic transmission. This figure was created with *BioRender*.

Finally, as in the case of other respiratory viruses with neurotropic potential, including the influenza A virus (68), SARS-CoV-2 could gain access to the CNS through the vagus nerve. The terminals of this nerve are located along the respiratory and gastrointestinal tracts, sites with high expression of ACE2 (49, 50) and enriched with ACE2+TMPRSS2+ enteric neurons (56). From these organs, the virus could gain access to the brain stem, taking advantage of the polarization of neurons and the machinery responsible for retrograde neuronal communication, or through endocytosis and clathrin-mediated exocytosis, as observed in the case of the transsynaptic transmission of the porcine coronavirus HEV 67N (69). The possible CNS invasion routes used by SARS-CoV-2 are illustrated in Figure 2.

## Neurological Manifestations of COVID-19 Due to CNS Involvement

### Non-specific Neurological Symptoms

Some of the initial descriptions of the clinical phenotype of patients infected with SARS-CoV-2 showed that up to 10% of individuals with COVID-19 manifested non-specific neurological symptoms such as headache and dizziness (5, 6, 70). In a more recent report by Mao et al., a third of patients with COVID-19 presented non-specific neurological manifestations, including dizziness (16.8%), headache (13.1%), loss of consciousness (7.5%), and seizures (0.5%) (10). Two recent systematic reviews and meta-analyses showed that headache and dizziness are among the most frequent neurological symptoms affecting COVID-19 patients (71, 72). Interestingly, headache can occur even in the absence of fever and can be manifested as a migraine, tension-type, or cluster headache (73).

These non-specific neurological symptoms may reflect the neurotoxic effect of hypoxemia and the cytokine storm observed in patients with severe COVID-19. However, some of these manifestations must be differentiated from delirium, and other secondary causes, such as metabolic, gastrointestinal, renal, and hematological complications, must be ruled out, particularly in aged patients with underlying comorbidities. Interestingly, non-specific neurological manifestations are more frequent in patients who progress to respiratory failure, which supports the hypothesis about a possible contribution of the CNS infection to the respiratory failure caused by SARS-CoV-2 (67). Furthermore, patients with unspecific neurological symptoms have shown higher degrees of leukopenia, thrombocytopenia, and elevated blood urea nitrogen levels (BUN) (10). These data suggest that some of these findings might have certain prognostic value to predict the occurrence of more severe neurological complications. However, the predictive potential of non-specific neurological symptoms needs to be further evaluated in prospective studies. This would be of great help for the timely detection of patients at risk of neurologic sequela.

### COVID-19 Associated Meningitis, Encephalitis, and Acute Necrotizing Encephalopathy

Acute meningitis and encephalitis are dramatic complications of various viral infections that often result in high morbidity and mortality rates due to their severity. Coronaviruses have also been associated with these neurological complications in humans. For instance, the HCoV-OC43 coronavirus was isolated from the brain of a patient who died of viral encephalitis (74). Likewise, both SARS-CoV-1 (46) and MERS-CoV have been reported to cause encephalitis (75).

In this context, SARS-CoV-2 can also cause viral meningitis and encephalitis, as demonstrated by a recent report of a 64-year-old patient with laboratory-confirmed COVID-19 who presented neurologic manifestations during the infection, including lethargy, clonus, and pyramidal signs in the lower limbs as well as stiff neck and Brudzinski sign (76). The cerebrospinal fluid (CSF) study revealed high protein levels and hypoglycorrhachia, although the virus could not be isolated from CSF in this case. The patient received antiviral drugs and symptomatic measures, progressing favorably without neurological complications. Similarly, in another report, a 24-year-old man with a 3-day history of headache developed fever, loss of consciousness, and seizures (77). Upon hospital admission, he presented signs of meningeal irritation and a low Glasgow Coma Scale (GCS) score, requiring mechanical ventilation. During his medical follow-up, he continued presenting seizures resistant to pharmacological treatment. His laboratory studies revealed high protein levels and pleocytosis in the CSF, and the MRI showed hyperintensities in the mesial temporal lobe. The patient tested negative for COVID-19 when his nasopharyngeal swab specimen was analyzed. Nonetheless, the result of the test was positive in the CSF sample. This finding was the first demonstration of the presence of SARS-CoV-2 in the CNS. The occurrence of encephalitis as the initial and even only manifestation of COVID-19 has latter been reported in three additional cases from China and the United States (78–80). Finally, another case report showed that SARS-CoV-2 infection could cause acute necrotizing encephalopathy (81). This entity is a severe neurological complication resulting from the disruption of the BBB associated with the cytokine storm observed in individuals with a critical illness.

Together, these cases illustrate the development of severe acute neurological complications in patients with COVID-19, confirming the neurotropism of SARS-CoV-2. These findings justify the intentional search for SARS-CoV-2 infection in patients attending with acute neurological manifestations and febrile illness even in the absence of respiratory symptoms. The frequency and factors related to the severity of these complications need to be extensively investigated in future studies. This would require the conduction of comprehensive characterizations of genetic and immunological characteristics associated with the risk of severe neurological complications in COVID-19 patients. In addition, future studies evaluating the relationship between the temporal dynamics of neurological findings, the kinetics of viral loads in the circulation and CNS, and distinctive patterns of circulating immune mediators are warranted. This might inform about the potential of contagiousness of COVID-19 patients according to their neurological symptoms and reveal possible candidate biomarkers to distinguish individuals at high risk of severe neurologic sequela.

### Cerebrovascular Disease

The antecedent of a recent respiratory infection, such as influenza, or influenza-like illness, has been related to acute cardiovascular complications, including acute myocardial infarction and stroke (82). Among infectious causes of vascular events, the infection with the varicella-zoster virus (VSV) is the most frequent viral cause of stroke (83). The study by Mao et al. revealed that 3% of patients with COVID-19 also present stroke as their only neurological manifestation of the infection. From these, the majority were affected by ischemic strokes (six ischemic vs. one hemorrhagic from a total of 214 patients included in the study) (10). Stroke in individuals with COVID-19 occurs late during the disease and is more frequently observed among patients with severe respiratory failure. Interestingly, some COVID-19 patients were admitted to medical centers with hemiplegia and no history of respiratory symptoms (10). Other studies have also reported stroke in patients with severe COVID-19, even among young individuals (84, 85). This might indicate that stroke could be a result of vascular alterations directly driven by the virus, although the presence of cardiovascular risk factors can increase the incidence of this complication. A recent meta-analysis found that stroke, apart from being a frequent complication of COVID-19, has a considerable prognostic value to predict the risk of mortality. As such, patients with stroke have a 3-fold increase in the risk of death due to COVID-19 (86).

The association between stroke and COVID-19 might result from the critical condition and the pro-inflammatory state that prevails in most severely ill patients. The increased levels of pro-inflammatory cytokines in these individuals could alter the normal function of the cerebral vessels. These factors, along with the high prevalence of cardiovascular risk factors among patients with COVID-19, can trigger cerebrovascular complications. The endothelial infection of the cerebral vasculature due to its high expression of the ACE2 receptor might further contribute to these complications (49). In fact, the incidence of stroke in patients with VSV infection is a consequence of the invasion of the cerebral arteries by the virus (83). Other mechanisms by which SARS-CoV-2 could cause stroke include coagulation disorders. The abnormalities in the coagulation of severe COVID-19 patients have unique characteristics that partially resemble disseminated intravascular coagulation (DIC) or thrombotic microangiopathy. These alterations increase the risk of thrombotic complications and death due to COVID-19 (87). Acute myocarditis has been reported in patients with SARS-CoV-2 infection (88, 89). This cardiac complication could trigger events of brain embolization and stroke, which might explain the incidence of cerebral infarctions in young patients with COVID-19 in the absence of cardiovascular risk factors.

## Neurological Manifestations of COVID-19 Due to PNS Involvement

### Peripheral Neuropathy and Myopathy Associated With SARS-CoV-2 Infection

During the outbreak caused by SARS-CoV-1, some studies described the occurrence of peripheral motor neuropathy and myopathy among infected individuals, mostly as part of the spectrum of manifestations of the critical illness polyneuropathy and myopathy disorders (90). Similarly, 2.3% of patients with COVID-19 have presented neuropathic pain, probably associated with peripheral neuropathy, whereas 10.7% of the cases showed data of skeletal muscle injury with elevated serum levels of creatine phosphokinase (CPK) (10). As in the case of other neurological manifestations, neuropathic pain and myopathy have been more frequently observed in patients with severe forms of COVID-19. Notably, these symptoms occur earlier during the disease in individuals with SARS-CoV-2 infection as compared to patients affected by SARS-CoV-1 (10). This suggests that the neuropathy and myopathy of COVID-19 are directly associated with the injury of peripheral nerves and striated muscles driven by the virus. COVID-19 patients with neuropathy and myopathy, however, exhibit higher levels of neutrophils and acute phase reactants than individuals without neurological manifestations (10). The nerve and muscle disorders observed during SARS-CoV-2 infection might thus be associated with a critical illness polyneuropathy of rapid development due to the more deteriorated clinical condition of patients with COVID-19 as compared to cases of SARS-CoV-1 infection. Vascular, thrombotic, ischemic, and direct nerve and muscle alterations driven by SARS-CoV-2 can contribute to neuropathy and myopathy of COVID-19 patients.

### Cranial Neuropathies in Patients With COVID-19

A wide range of infectious diseases can cause complicate within cranial neuropathies like facial palsy and ophthalmoplegia. Among the pathogens that cause these manifestations with more frequency are HIV and VSV (91). Cranial nerves might also be susceptible to a direct or indirect injury caused by SARS-CoV-2. In fact, according to a recent study, about 85% and 88% of patients with COVID-19 develop olfactory and gustatory dysfunction (66). Similarly, in another investigation conducted in Italy, about 33% of patients with COVID-19 reported taste and/or olfactory disorders (92). These findings might be specific for SARS-CoV-2 infection and could predict the causative pathogen in patients with acute respiratory illness. Indeed, smell and/or taste disorders were more frequently observed among COVID-19 patients as compared to individuals with laboratory-confirmed influenza infection in a case-control study conducted in Spain (93). These observations might be of particular relevance for the upcoming flu season, which is predicted to be historically unique due to the convergence of influenza and COVID-19. During such an envisioned scenario, the differentiation of these diseases by clinical manifestations could be complicated. Nonetheless, the correct identification of the causative pathogen have therapeutic implications, such as the selection of adequate antiviral treatment. The presence of smell and taste dysfunction could thus be useful to distinguish COVID-19 from influenza. Furthermore, these symptoms can precede the onset of respiratory symptoms (94, 95), and their presence may predict a milder clinical course of the disease (96). Notably, COVID-19-related olfactory dysfunction has not been associated with rhinorrhea or nasal congestion, and more than half of the affected patients did not recover the function of the olfactory nerve (66). This suggests direct damage to the olfactory epithelium, which further reaffirms the possibility of an entry route of SARS-CoV-2 through the cribriform plate, reaching the olfactory bulb from where it spreads to other parts of the CNS (62).

## Autoimmune Neurological Syndromes Associated With COVID-19

### Guillain-Barré Syndrome

The association between viral infections and Guillain-Barré syndrome has been largely described in the past (97). Infection with *Campylobacter jejuni*, cytomegalovirus, or *Mycoplasma pneumoniae* precedes Guillain-Barré syndrome in up to two-thirds of affected individuals (98). More recently, several viral emerging diseases have shown this syndrome as one of its more common and severe complications, as is the case of the infection with the Zika virus (99). Although this association was not described in patients infected with SARS-CoV-1, cases of Guillain-Barré syndrome were observed during the outbreak of MERS-CoV (100). In this context, the infection with SARS-CoV-2 could also complicate with or manifests as Guillain-Barré syndrome. In a recent report, Zhao et al. described the case of a woman that developed asymmetric and progressive muscle weakness in the lower limbs, associated with leukopenia and high protein levels in the CSF without pleocytosis, accompanied by data of demyelinating neuropathy in studies of nerve conduction (101). The patient had a history of a recent trip to the city of Wuhan, and she showed no evidence of respiratory system involvement at the time of symptom onset. A total of 8-days later, the appearance of fever, respiratory symptoms, and radiological data compatible with pneumonia was documented, confirming the SARS-CoV-2 infection by laboratory testing in a nasopharyngeal swab sample. The patient received intravenous immunoglobulin treatment recovering the neurological function without sequel. Similarly, in another case from Italy, a 71-year-old male also developed Guillain-Barré syndrome before the onset of any respiratory symptom (102).

These reports suggest that Guillain-Barré syndrome may be the first manifestation of COVID-19, presenting as a para-infectious phenomenon. This is due to the parallel occurrence of neurological and respiratory symptoms, instead of the postinfectious pattern observed in other infections, including the MERS-CoV (100). Nonetheless, two new case reports of six COVID-19 patients demonstrated that Guillain-Barré syndrome could occur a few days after the onset of respiratory symptoms (103, 104). Collectively, these findings obligate physicians to continuously monitor the neurological condition of patients with suspected or confirmed SARS-CoV-2 infection to identify any sign of demyelinating neuropathy. Furthermore, the occurrence of Guillain-Barré syndrome associated with COVID-19 must be carefully distinguished from the myopathy and neuropathy disorders of the critically ill patient.

### Miller Fisher Syndrome

Miller Fisher syndrome is a rare neurological disease that is considered a variant of the Guillain-Barré syndrome. This disorder is characterized by the triad of abnormal muscle coordination, paralysis of the eye muscles, and the absence of tendon reflexes. Miller Fisher syndrome can be associated with a history of recent viral illness; however, no evidence exists of its association with human coronavirus infections. Interestingly, a recent paper has reported the occurrence of the triad of ataxia, areflexia, and ophthalmoplegia in a 50-year-old man that presented cough, fever, anosmia, and hypogeusia some days before the onset of the neurological symptoms. The infection with the SARS-CoV-2 infection was confirmed in an oropharyngeal swab sample, and blood tests showed lymphopenia, elevated C-reactive protein levels, and anti- GD1b-IgG antibodies. He received treatment with intravenous immunoglobulin, which caused significant improvement in his neurological functions. Collectively, the literature summarized here indicates that neurological syndromes caused by aberrant immune responses, such as Guillain-Barré and Miller Fisher syndromes, can occur as part of the clinical spectrum of SARS-CoV-2 infection. Despite this, the immune mechanisms implicated in these phenomena, and the possibility of a direct neuropathic effect driven by the virus are unknown.

Molecular mimicry has been proposed as the primary immune alteration underlying the development of Guillain-Barré syndrome during infections (105). This phenomenon is related to the presence of carbohydrates in infectious agents of similar structural characteristics as compared to carbohydrates expressed on neuronal membrane ganglioside and galactocerebrosides. For instance, the lipopolysaccharides of *Campylobacter jejuni* share ganglioside-like epitopes with peripheral nerves (106), whereas galactocerebroside-like structures are present in glycolipids of *Mycoplasma pneumoniae* (107). These molecular similarities trigger the production of anti-glycolipid antibodies, which mediate autoimmune damage to peripheral nerves. Ganglioside- or galactocerebroside-like epitopes have not been described in SARS-CoV-2. However, the S protein of this virus possesses 22 N-linked glycan sequons per promoter (20), that could potentially share certain similitude with carbohydrates localized on the surface of the host nerve cells. Future studies are required to evaluate the serologic features of anti-glycolipid antibodies in patients with COVID-19 to elucidate possible mechanisms underlying the association between SARS-CoV-2 infection and Guillain-Barré syndrome.

## Neuropsychiatric Manifestations of COVID-19

Several viruses affecting humans have been implicated in the development of psychiatric symptoms due to their neurotropism. The immediate antecedent for the global transmission of a respiratory pathogen was the 2009 pandemic associated with the emergence of a novel influenza A (H1N1) virus. Several neuropsychiatric manifestations during the outbreak of influenza were observed among infected patients, including fear and behavioral changes (108). Similarly, during the SARS-CoV-1, a range of psychiatric disorders was identified, including anxiety, depression, suicidal ideation, and hallucinations (109, 110). A recent systematic review found a high incidence of confusion, depression, anxiety, memory impairment, insomnia, and steroid-induced psychosis among patients with SARS-CoV-1 or MERS-CoV infection (111). The mechanisms underlying psychiatric disorders in patients with viral infections are not precise, but they might be related to the structural and functional disruption of the BBB mediated by circulating inflammatory cytokines produced in response to viruses. These mediators might also alter neuronal networks implicated in cognitive functions. Indeed, several psychiatric illnesses, including schizophrenia, have been proposed to result from immune-mediated pathogenic mechanisms (112).

In this sense, it is clear that the current pandemic can cause indirect effects on the mental health of infected and non-infected people due to quarantine and social distancing measures, which constitute sources of distress additional to the daily life problems (113). At this moment, however, there is little literature about neuropsychiatric disorders directly associated with the infection with SARS-CoV-2. In a case series of three patients with laboratory-confirmed COVID-19 and no evidence of respiratory symptoms, it was found that such individuals presented anxiety, agitation, paranoid behavior, disorganized thinking, and auditory hallucinations (114). Other authors have also reported that delirium can be present in a high percentage of COVID-19 patients (111). Thus, as mentioned before, delirium must also be considered in the differential diagnosis of individuals with acute neuropsychiatric manifestations associated with SARS-CoV-2 infection. Finally, the virus could lead to long-term neuropsychiatric and cognitive sequels. In fact, survivors of SARS-CoV-1 and MERS-CoV have been found to present depression, anxiety, and post-traumatic distress syndrome several months after the diagnosis (111). Therefore, it is essential to conduct long prospective observational studies to estimate the incidence of psychiatric disorders in the post-illness stage of COVID-19. Future studies must address possible links between the antecedent of SARS-CoV-2 infection and the incidence of chronic neurodegenerative disorders. The studies about the spectrum of neurological and psychiatric manifestations of COVID-19 are summarized in Table 1.

**Table 1:** The spectrum of neurological manifestations of SARS-CoV-2 infection.

**Clinical finding**	**No. of patients**	**Author**	**Country**	**References**
Headache	3/39	Huang et al.	China	(5)
	9/138	Wang et al.	China	(6)
	8/99	Chen et al.	China	(70)
	28/214	Mao et al.	China	(10)
	29/112	Porta-Etessam et al.	Spain	(73)
Dizziness	13/138	Wang et al.	China	(6)
	36/214	Mao et al.	China	(10)
Loss of consciousness/altered mental status	9/99	Chen et al.	China	(70)
	16/214	Mao et al.	China	(10)
Seizures	1/214	Mao et al.	China	(10)
Cerebrovascular disease	6/214	Mao et al.	China	(10)
	3/58	Helms et al.	France	(84)
	5 cases	Oxley et al.	United States	(85)
Meningitis/encephalitis	1 case report	Moriguchi et al.	Japan	(77)
	1 case report	Yin et al.	China	(76)
	1 case report	Duong et al.	United States	(78)
	1 case report	Filatov et al.	United States	(79)
	1 case report	Ye et al.	China	(80)
Olfactory and/or taste dysfunction	20/59	Giacomelli et al.	Italy	(92)
	31/79	Beltrán-Corbellini et al.	Spain	(93)
	128/169	Yan et al.	United States	(96)
	130/374	Spinato et al.	Italy	(95)
	357/417	Lechien et al.	Belgium	(66)
	23/214	Mao et al.	China	(10)
	2 cases	Lorenzo Villalba et al.	Spain	(94)
Peripheral neuropathy/nerve pain	5/214	Mao et al.	China	(10)
Myopathy	23/214	Mao et al.	China	(10)
Guillain-Barre syndrome	1 case report	Zhao et al.	China	(101)
	1 case report	Alberti et al.	Italy	(102)
	1 case report	Sedaghat et al.	Iran	(103)
	5 cases	Toscano et al.	Italy	(104)
Miller-Fisher syndrome	1 case report	Gutierrez-Ortiz et al.	Spain	(115)
Neuropsychiatric manifestations	3 cases	Ferrando et al.	United States	(114)

*Other neurological manifestations reported in COVID-19 patients include ataxia, acute hemorrhagic necrotizing encephalopathy, polyneuritis cranialis, and neuralgia (10, 81)*.

## Potential Therapeutics for the Neurological Complications of SARS-CoV-2 Infection

Supportive measures, along with strict control and prevention of fever, high blood pressure, elevated glucose, and seizures, may ameliorate the neurotoxic potential of SARS-CoV-2 infection. Currently, there are no mechanism-based therapeutics specific for neurological complications of COVID-19. The evidence curated in this review suggests possible pathologic processes underlying the involvement of the CNS/PNS during SARS-CoV-2 infection. A better understanding of these mechanisms may reveal targets for therapeutic interventions. Direct effects driven by the virus, such as the infection of brain blood vessels and nerve cells, are proposed to play a role in neurologic manifestations of COVID-19. Although studies addressing the relationship between viral loads in the CNS and the severity of such manifestations are required, reducing the number of copies of SARS-CoV-2 in the circulation and tissues might be a useful strategy. Remdesivir is the only antiviral drug with demonstrated capacity to blocking SARS-CoV-2 replication in pre-clinical trials approved for usage in humans. This antiviral drug reduces the time of clinical recovery in patients with COVID-19 (116). The benefits of remdesivir for patients with neurological complications have not been evaluated. Other candidate agents that antagonize the activity of the host proteases implicated in the infection process of SARS-CoV-2 may be useful to limit the infective capacity of this virus. These include the camostat mesylate and nafamostat mesylate, two compounds that block the activity of TMPRSS2 (19, 117). The effects of these drugs on the severity and recovery of neurologic sequela associated with COVID-19 must be evaluated in future clinical trials. Passive immunization by transfer of convalescent human plasma may also contribute to reduce the viral loads and prevent or reverse the development of neurological symptoms. This assumption is supported by recent systematic reviews that found that convalescent plasma therapy improves symptoms, reduce viral loads, and diminishes mortality in COVID-19 patients (118). The time at which these proposed interventions would be more useful to counteract neurologic sequela of COVID-19 is unknown.

Immune-mediated neurotoxicity is an obvious therapeutic target for individuals with SARS-CoV-2 infection (59). The immune profile of patients with severe COVID-19 resembles the cytokine release syndrome (CRS) observed after CAR-T-cell therapy (34, 35). As aforementioned, individuals receiving CAR-T cells who develop CRS are at risk of injury to the nervous system. Therefore, lessons from the treatment of patients with CAR-T-cell-therapy-associated neurotoxicity might be applicable to COVID-19 patients. Tocilizumab, an anti-IL-6 monoclonal antibody, is the primary treatment for CRS (119), and is currently being used to ameliorate inflammatory manifestations caused by SARS-CoV-2 infection (120). In patients with severe COVID-19, tocilizumab declined cytokine production and reduced the risk of intubation requirement (120). However, the utility of tocilizumab for management of CRS-associated neurotoxicity is controversial (121). Interestingly, mouse models have revealed that the antagonist of the receptor of IL-1β anakinra might be a better option than tocilizumab for the treatment of CRS and neurotoxicity after CAR-T-cell therapy (122). As a strong induction of IL-1β has been observed in severely ill COVID-19 patients (32), the potential use of anakinra deserves further investigation. Monocytes and macrophages also contribute to the development of CRS and neurotoxicity after CAR-T-cell therapy. As such, inhibition of GM-CSF with monoclonal antibodies has shown to reduce neuroinflammation in Phase I studies of patients receiving CAR-T-cell therapy (123). Interestingly, in a recent study conducted in patients with COVID-19, the GM-CSF blockade with mavrilimumab improved clinical symptoms, survival, and reduced intubation requirement (124). Inhibition of GM-CSF is thus a potential therapeutic for patients with COVID-19 that develop neurological complications. Short courses of steroids are safe and provide some benefits for the treatment of immune-mediated neurotoxicity. Specifically, dexamethasone may constitute a good candidate due to its excellent CNS penetration and beneficial effect on the integrity of the BBB. Dexamethasone has been safely used in patients with acute respiratory distress syndrome, reducing the requirement of mechanical ventilation and mortality (125). These data may support the use of dexamethasone for the management of neurological manifestations of SARS-CoV-2 infection, although possible consequences of the dexamethasone-induced immunosuppression need to be evaluated.

The breaching of the BBB is an important event for the development of neurotoxicity in COVID-19 patients and individuals under CAR-T-cell therapy. Pharmacological agents targeting the BBB may thus be useful to treat severe neurological manifestations of COVID-19. The sonic hedgehog (SHH) signaling pathway is essential for the maintenance of integrity and immune homeostasis of the BBB (126). Agonists of SHH, such as purmorphamine, a compound that activates the SHH by promoting smoothened protein (127), reduce BBB damage in animal models of infection and ischemic stroke (128–130). Although little evidence exists of the safety of purmorphamine in humans, this agent warrants further exploration for the management of neurotoxicity associated with severe infections, including COVID-19.

## Implications of COVID-19 Pandemic for the Neurology Field

The magnitude of the current pandemic has generated concerns among professionals from various areas of medicine. The evidence summarized in this review shows that neurology is an active area of medicine at the frontline of the current pandemic. Actually, due to the increasing number of confirmed cases around the world and the neurotropic potential of SARS-CoV-2, it is highly likely that neurologists would have to provide medical care to patients with COVID-19, some of which might present neurological manifestations. The first and most important precautionary measure that must be taken by neurological centers around the world is to improve the knowledge of the disease among health care professionals. Obviously, measures of personal protection and social distancing should be extreme in neurology and neurosurgery clinics since it is possible that some COVID-19 patients attending with neurological symptoms with a not yet confirmed SARS-CoV-2 infection may constitute potential foci of inadvertent contagion for doctors, especially if they do not manifest respiratory symptoms initially. To prevent contagions, it is important to investigate previous contact with people with laboratory-confirmed COVID-19 in patients attending to neurological centers. Similarly, it is crucial to maintain a high degree of clinical suspicion about possible SARS-CoV-2 infection in patients presenting an acute neurological condition. Furthermore, the continuous surveillance and intentional search for neurological complications in patients with confirmed SARS-CoV-2 infection are necessary and even mandatory because these measures could allow the timely establishment of therapeutic strategies for limiting neurologic sequelae.

Finally, the current pandemic may obligate some changes in normal care to patients with neurologic disorders at medical centers receiving many COVID-19 cases. Of particular importance is the possible impact of this pandemic in the triage and management of patients with stroke and other acute neurological emergencies due to resource re-allocation. Also, the interruption of activities at many neurology outpatient clinics may affect the management of chronic neurological conditions, requiring increased usage of tools such as telemedicine and e-care.

## Conclusions

The clinical phenotype of COVID-19 encompasses a spectrum of neurological manifestations of varying severity that, besides respiratory symptoms, can cause high morbidity and mortality rates in individuals with SARS-CoV-2 infection. The current review constitutes a useful reference to improve our understanding of the pathophysiological mechanisms of SARS-CoV-2 infection and should motivate further studies about novel strategies to mitigate the impact of the current pandemic on the field of neurology.

## Author Contributions

PG-O, JC-P, and CS-M drafted the manuscript. FP-S, AR-N, and GG-Q are medical students from the Centro Especializado en Neurocirugía y Neurociencias México (CENNM) and the Escuela Nacional de Medicina y Homeopatía, Instituto Politécnico Nacional in Mexico City. They participated in the search for scientific literature and revised the paper for intellectual content. All authors read and approved the final version of the manuscript.

## Conflict of Interest

The authors declare that the research was conducted in the absence of any commercial or financial relationships that could be construed as a potential conflict of interest.
